# Puerperal Fever

**Published:** 1912-01

**Authors:** 


### Puerperal Fever.

[FROM BRYCE'S POCKET PRACTICE.]

Puerperal fever is due to the infection of the puerperal uterus by careless persons, whose dirty hands carry infective micro-organisms into the genital tract of the newly-delivered woman. It is therefore prevented by strict aseptic cleanliness of the woman, and her doctor, and nurse. Many micro-organisms have been found acting here, but the most common one is the streptococcus pyogenes, the diplococcus of erysipelas. The lowering of vital resistance renders some women more susceptible to the attacks than others. The gonococcus is now recognized as a cause of many cases.

The attack occurs some days after delivery, beginning with a chill, followed by fever. Sometimes the infection is so deadly that the patient dies before the fever has time to develop, from intense toxemia. The pulse is faster than usual in proportion to the fever. Respiration rises to 60. The patient shows great depression. Headache is always present, and usually severe. The tongue is coated, the breath foul, nausea is common, the stomach tender, bowels constipated, soon becoming loose, the stools very offensive, and there is a little jaundice. Perspiration may be free. The milk is scanty or ceases. The face is anxious and the skin dusky. The spleen is enlarged. The lochia becomes offensive and scanty or suppressed; the uterus tender, soft, and relaxed; the abdomen distended.

Puerperal sepsis may be prevented by careful treatment of the patient during pregnancy, and asepsis during confinement. Keep up the blood with iron, keep the bowels clear and clean, see that albuminuria is dissipated by diuretics and especially buttermilk, and try to keep the patient in the best possible state, physically and mentally.

When labor begins, flush the vagina with non-toxic antiseptic solutions; if gonorrhea is present, saturate with calx sulphurata, and compel nurses to have their hands aseptic. Don't forget your own, and have every instrument and dressing free from disease germs. Flush the rectum and colon thoroughly. The secundines should be removed completely, though they do no harm unless the germs are conveyed to them from without, or absorbed from the bowel. Do not use vaginal douches after delivery unless they are really needed. Fetid lochia, as a rule, call for them, but the most deadly infections may not be attended by fetor of the discharges. Dorland recommends zinc sulphocarbolate, two to three drams to the pint, for cleansing vaginal douches. Firm uterine contraction should be secured by a sufficient dose of ergot.

If the malady commences, the uterus and vagina should at once be thoroughly cleaned out and disinfected by irrigation and the curette, with thorough asepsis of hands and instruments, of the vulva, the vagina, and the uterine cavity, the latter by curette, douche, and a suppository of iodoform, fifteen to thirty grains.

As systemic germicides, the remarkable control exerted by pilocarpine over sthenic erysipelas indicates its value here when the same germ is in operation, and the same may be said of calx sulphurata in gonorrhea. Give either to full effect. Quinine is a broken reed here. Quell excessive fever by the use of aconitine and digitalin, aided by veratrine if the pulse demands, or strengthened by strychnine if debility is foreseen. Flush the bowels with small, repeated doses of salines, and similar enemas. Subcutaneous injections of saline solution are useful. The diet should be of fluids in small quantities, given every two hours—clam broth, coffee, raw white of an egg. Pain may require morphine. Treat the symptoms, sustain the heart, keep up renal activity, and quiet restlessless with gelseminine. Puerperal fever is a malady we know how to prevent, and how to treat, and the prognosis is not nearly as bad as it was fifty years ago.—*Southern Clinic,* Richmond, Va.

---

In case of urinary extravasation from rupture of the urethra, make no attempt to pass a catheter, and waste no time in incising the edematous area, but perform perineal urethrotomy at once.—*American Journal of Surgery.*

